# Does the placement of acetabular cups differ between right and left sides for a right-handed surgeon operating through a direct lateral approach? A comparative study

**DOI:** 10.1186/s42836-024-00278-8

**Published:** 2024-11-05

**Authors:** Ahmed A. Khalifa, Ahmed M. Abdelaal

**Affiliations:** 1 Orthopaedic Department, Qena University Hospital, Qena, 83523 Egypt; 2 Department of Orthopedic Surgery and Traumatology, Assiut University Hospital, Assiut, 71515 Egypt

**Keywords:** Surgeon handedness, Acetabular cup position, Safe zone, Inclination, Anteversion

## Abstract

**Purpose:**

Although many factors were suggested to affect acetabular cup positioning during primary total hip arthroplasty, the effect of surgeon handedness was rarely evaluated. We aimed primarily to assess the difference in cup positioning (inclination and anteversion) between the right and left sides during primary THA. Secondly, to check the difference in the percentages of cups positioned in the safe zone for inclination and anteversion and if there will be a difference in cup positioning according to the type of cup fixation (cemented vs. cementless).

**Methods:**

Cup inclination and anteversion of 420 THAs were radiographically evaluated retrospectively. THAs were performed by a senior right-handed surgeon, who operated through a direct lateral approach in a lateral decubitus position using manual instruments and freehand technique for cup placement. Patients were assigned to two groups: Group A (right, or dominant side), and Group B (left, or non-dominant side), with equal cases of THAs (*n* = 210) in each group.

**Results:**

No difference was found in patients’ basic characteristics, preoperative diagnosis, and cup fixation (54.3% cemented and 45.7% cementless) between the two groups. There was a significant difference in cup inclination between Groups A and Group B (40.1° ± 6.3° vs. 38.2° ± 6.1°) (*P* = 0.002). No significant difference was revealed in anteversion between the two groups (11.7° ± 4.4° vs. 11.8° ± 4.7°) (*P* = 0.95). The percentage of cups located within the safe zone in terms of both inclination and anteversion was 85.2% vs. 83.8% and 69% vs. 73.3% for Group A and Group B, according to Lewinnek and Callahan’s safe zones, respectively. There existed a significant difference in the cemented cup inclination between Group A and Group B (40.8° ± 6.4° vs. 38.3° ± 6.3°) (*P* = 0.004).

**Conclusion:**

Cup inclination is affected by the surgeon’s handedness when operating through a direct lateral approach and using a freehand technique, while anteversion is less affected. Furthermore, the difference is greater with cemented cups.

## Introduction

Total hip arthroplasty (THA) is one of the most successful surgical procedures [1]. However, its outcomes vary and could be affected by many factors, including but not limited to component positioning during surgery, which was proved to affect the function as well as the survival of implants [2–5].

Factors affecting component placement, particularly the acetabular cup, during THA, are extensively studied in the literature. These factors can be categorized into surgeon-, patient-, and surgery-related ones, including the patient’s body habitus (such as obesity) [6], patient’s position (lateral or supine) [7, 8], surgical approaches [9, 10], surgeon experience and learning curve [11, 12], and pelvic tilt and spine-pelvic relationship [13, 14], among others. However, in most cases, more than one factor is in play [5, 6, 11, 12, 14–16].

The effect of surgeon handedness on component placement during joint replacement surgeries was scarcely reported in the literature. Few studies examined its effect during knee arthroplasty surgery, including total and unicompartmental knee arthroplasties (TKA and UKA [17–19]. Furthermore, surgeon handedness and its effect on appropriate cup placement were documented as contributing factors in some THA studies, and most of these studies included experienced right-handed (RHD) surgeons operating through a posterolateral approach and rarely the direct lateral approach, mostly using cementless implants [20–24].

So, the primary objective of the current study was to assess the difference in cup positioning (inclination and anteversion) between the right and left sides during primary THA performed by an RHD surgeon operating through a direct lateral approach. The secondary objectives were to look at the difference in the percentages of cups positioned in the safe zone for inclination and anteversion and to see if there is a difference in cup positioning by the type of cup fixation (cemented vs. cementless).

We hypothesized that there would be a difference in cup positioning between the dominant (right) and non-dominant (left) sides; more cups would be placed within the safe zones on the dominant side, and the type of fixation would not make a difference.

## Patients and methods

The current study was a retrospective radiological observational study. The authors affirmed that this work followed The Code of Ethics of the World Medical Association (Declaration of Helsinki), and the ethical committee of our institution waived the approval due to the study’s retrospective nature and as it did not involve any experimental maneuvers. We evaluated all the radiographs taken at the first postoperative visit (which was either one week postoperatively during the first surgical wound check or two weeks postoperatively during suture removal) of all patients who underwent primary THA between January 2021 and December 2022 performed by a senior surgeon who is an RHD and has over 20 years of experience. We included patients with adequate pelvis anteroposterior plain radiographs (evaluated by a radiology consultant) when surgery was considered as a simple primary THA (cemented or cementless) who were diagnosed preoperatively as having primary osteoarthritis (OA), avascular necrosis (AVN), rheumatoid arthritis (RA), and femoral neck fracture (FNF). Patients receiving complex primary THA (such as THA performed for post-traumatic OA, acetabular defects, and acetabular dysplasia) or revision THA, or having inadequate radiographs were excluded from the study.

**Fig. 1:**
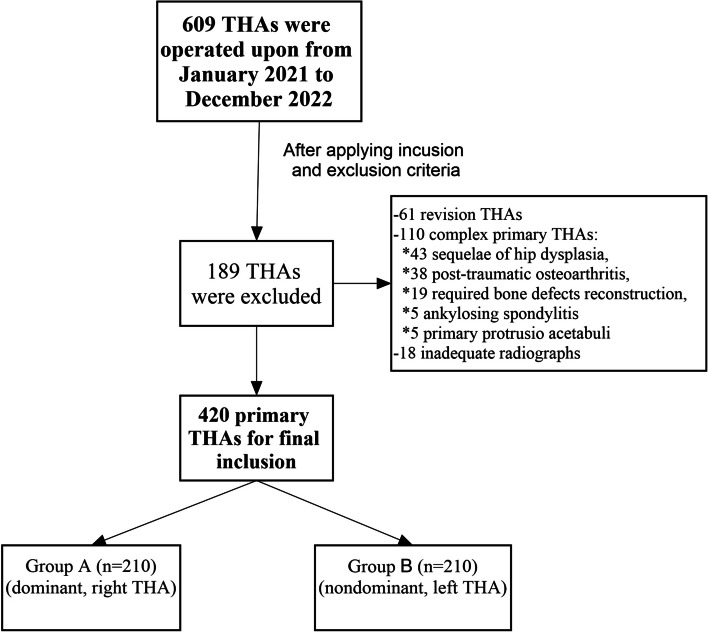
Flowchart of patient inclusion and assignment

To determine the sample size that ensured adequate power, we used the G*Power software (version 3.1.9.7.) for detecting a cup inclination difference between both sides of two degrees with a standard deviation up to five degrees [20]. The result showed that to achieve a power of 90% and an alpha level of 0.05, a minimum of 199 patients per group was required. Of 609 THA performed during the study period, 420 were eligible for inclusion and were equally assigned to the right (dominant, Group A, 210 THAs) and left (non-dominant, Group B, 210 THAs) side groups (Fig. 1).

### Surgical technique

**Fig. 2:**
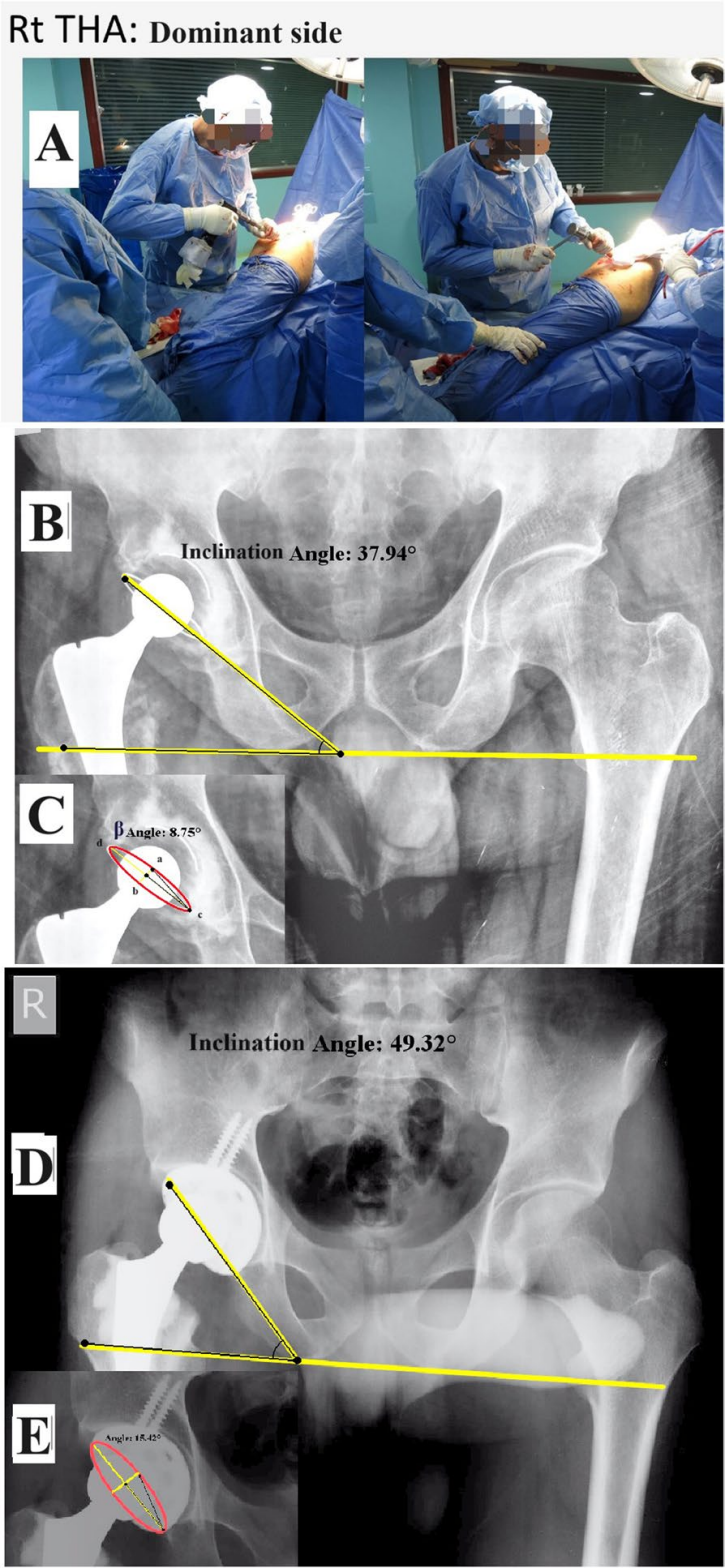
Performing THA on the right (dominant) side. **A**: intraoperative images showing the surgeon’s position. Cup inclination and anteversion assessment: **B** and **C**: a cemented THA. **D** and **E**: a cementless THA

**Fig. 3:**
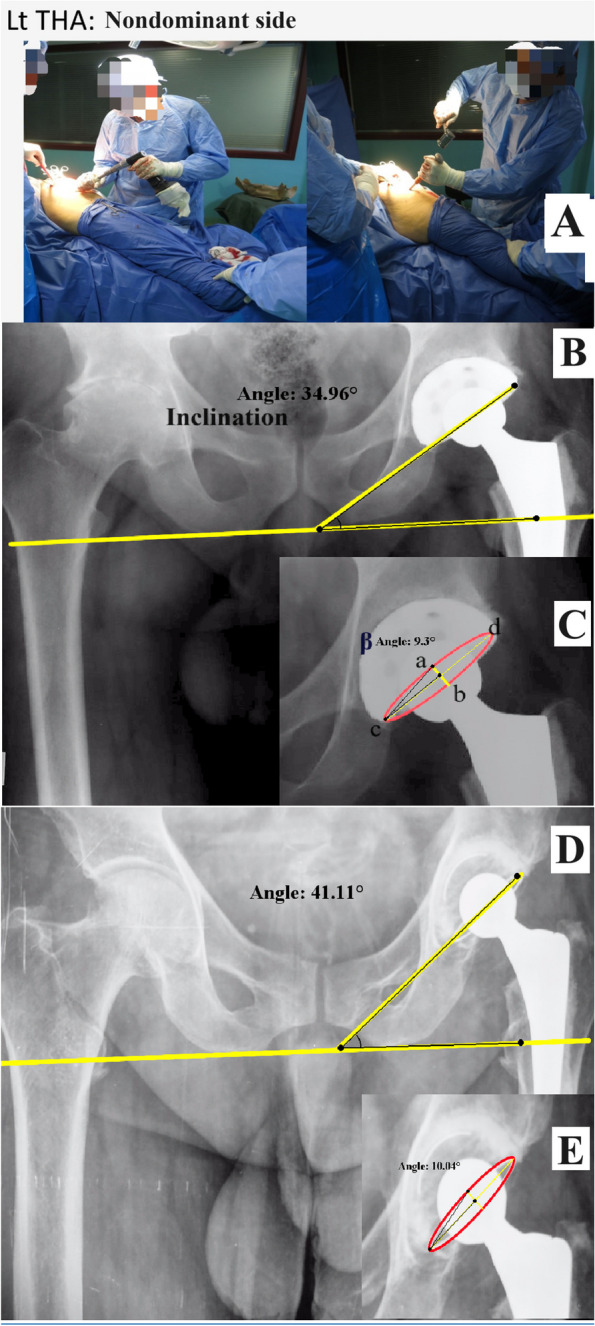
Performing THA on the right (dominant) side. **A**: intraoperative images showing the surgeon's position, cup inclination, and anteversion assessment: **B** and **C**: a cementless THA. **D** and **E**: a cemented THA

All patients were operated on under spinal anesthesia (unless the anesthesiologist advised to use general anesthesia) in a lateral decubitus position while the operative table was parallel to the floor. The operating surgeon constantly checked the position before draping, and two supports were placed to hold the pelvis (one posteriorly at the sacral level and the other over the symphysis pubis). All surgeries were performed through a modified direct lateral approach while the surgeon stood behind the patient. Cup positioning was performed using manual instruments and the freehand technique (no assistive technologies were used in any case). The surgeon aimed at an inclination of 40 ± 5 degrees and an anteversion of 15 ± 5 degrees. The inclination was adjusted by surgeons’ visual evaluation as referenced to the floor level, while the anteversion was adjusted relying on the transverse acetabular ligament (TAL) serving as an anatomical landmark [25, 26]. For preparing the acetabulum, the surgeon used his right hand to lead the instruments (reamering and hammering on the cup inserter), while the left hand was used to hold and support the instruments (Figs. 2A and 3A).

Choice between a cemented or cementless acetabular cup was mainly determined based on patient factors, such as age and acetabular bone quality (Cemented cups were preferred for patients with soft bone). Furthermore, the decision could be changed intraoperatively [27–30].

### Assessment

The cup inclination was measured as the angle between the trans-ischial line and a line connecting the cup’s most medial and lateral points (cup axis) (Figs. 2B, D and 3B, D). The anteversion was calculated using the method proposed by Liaw et al. (Anteversion = sin^−1^ tan β), where β is the angle between two lines (ac and cb, Figs. 2C, E and 3C, E) [31].

We determined the percentage of cups positioned within the safe zone as proposed by Lewinnek et al. [16] (inclination of 40 ± 10 degrees and anteversion of 15 ± 10 degrees) and Callanan et al., who lowered the upper inclination limit to 45 degrees [15].

To evaluate inter-observer agreement, each assessor measured parameters of 100 THAs on each side. We calculated the Intra-class Correlation Coefficient (ICC) to assess the consistency between the two observers. The ICC was 0.905 for cup inclination (SE 0.021, 95% CI: 0.864 to 0.945) and 0.809 for cup anteversion (SE 0.029, 95% CI: 0.778 to 0.859), indicating excellent agreement for cup inclination and good agreement for cup anteversion. According to the Edinburgh Handedness Inventory, the surgeon was confirmed to be RHD [32].

### Statistical analysis

Data were analyzed by using the Statistical Package for Social Science (SPSS), version 26.0 for Windows. Qualitative data were expressed as frequency and percent, and quantitative data were tested for normality by employing the Shapiro–Wilk test and presented as mean ± SD and range. Independent Sample T-test was conducted to compare the difference in inclination and anteversion between the right and left sides and between types of cup fixation. The Chi-square test was utilized to compare proportions between groups. The level of significance was set at a *P* value < 0.05.

## Results

Hips were assigned to each group in equal numbers, with each group having 210 hips. There was no difference in patients’ basic characteristics, preoperative diagnosis, and type of cup fixation, except that, in Group B, slightly more cemented cups were implanted than cementless ones (Table 1).

**Table 1:** Basic characteristics of included patients

		**Group A (*n* = 210)** **(dominant, right THA)**	**Group B (*n* = 210)** **(nondominant, left THA)**	***P*-Value**
**Age**		48 ± 13.3 (23–76)	50.4 ± 12.3 (23–75)	0.065*
**Sex**				
▪ Men		107 (51.0%)	113 (53.8%)	0.589†
▪ Women		103 (49.0%)	97 (46.2%)	
**BMI**		24.5 ± 2.9 (19.1–34.3)	23.9 ± 3.4 (18.7–33.2)	0.231*
**Diagnosis**				
▪ AVN		73 (34.8%)	57 (27.1%)	0.210†
▪ RA		50 (23.8%)	45 (21.4%)	
▪ FNF		48 (22.9%)	61 (29.0%)	
▪ OA		39 (18.6%)	47 (22.4%)	
**Types of cup fixation**				
	Total			
Cemented	228 (54.3%)	109 (51.9%)	119 (56.7%)	0.327†
Cementless	192 (45.7%)	101 (48.1%)	91 (43.3%)	
P-Value**	0.079	0.581	0.053	

Data were expressed as frequency and % or mean ± SD (range); BMI: body mass index, AVN: avascular necrosis, RA: rheumatoid arthritis, FNF: fracture neck of the femur, OA: osteoarthritis

^*^Independent Sample T-test compares the mean difference between both groups

^†^Chi-square test compares proportions between both groups

^**^Chi-square test compares proportions within Group A and Group B separately

The mean cup inclination was significantly higher in Group A than in Group B (40.1° ± 6.3° vs. 38.2° ± 6.1°) (*P* = 0.002), with an absolute difference of 1.9 degrees of mean inclination (range of differences between the right and left sides was from − 21.4 to 22.8 degrees). No significant difference was found in the mean anteversion between Group B and Group A (11.7° ± 4.4° vs. 11.8° ± 4.7°) (*P* = 0.95) (range of differences between the right and left sides was from −16.6 to 19 degrees) (Table 2).

**Table 2:** Comparison of cup positioning (inclination and anteversion) between both sides

	**Group A (*n* = 210)** **(dominant, right THA)**	**Group B (*n* = 210)** **(nondominant, left THA)**	***P*-Value**
***Inclination (in degrees)***			
**Mean ± SD**	40.1 ± 6.3 (21.8–60.3)	38.2 ± 6.1 (23.4–63.4)	0.002*
**Lewinnek et al**			
▪ Within safe zone (30–50)	189 (90.0%)	187 (89.0%)	0.750**
▪ Outside safe zone	21 (10.0%)	23 (11.0%)	
**Callanan et al**			
▪ Within safe zone (30–45)	155 (73.8%)	164 (78.1%)	0.304
▪ Outside safe zone	55 (26.2%)	46 (21.9%)	
***Anteversion (in degrees)***			
**Mean ± SD**	11.7 ± 4.4 (2.0–26.8)	11.8 ± 4.7 (2.0–27.1)	0.950*
▪ Within safe zone (5–25)	198 (94.3%)	194 (92.4%)	0.434**
▪ Outside safe zone	12 (5.7%)	16 (7.6%)	

Data were expressed as frequency and % or mean ± SD (range); Bold *P*-value indicates statistically significant

^*^Independent Sample T-test compares the mean difference between both groups

^**^Chi-square test compares proportions between both groups

**Fig. 4:**
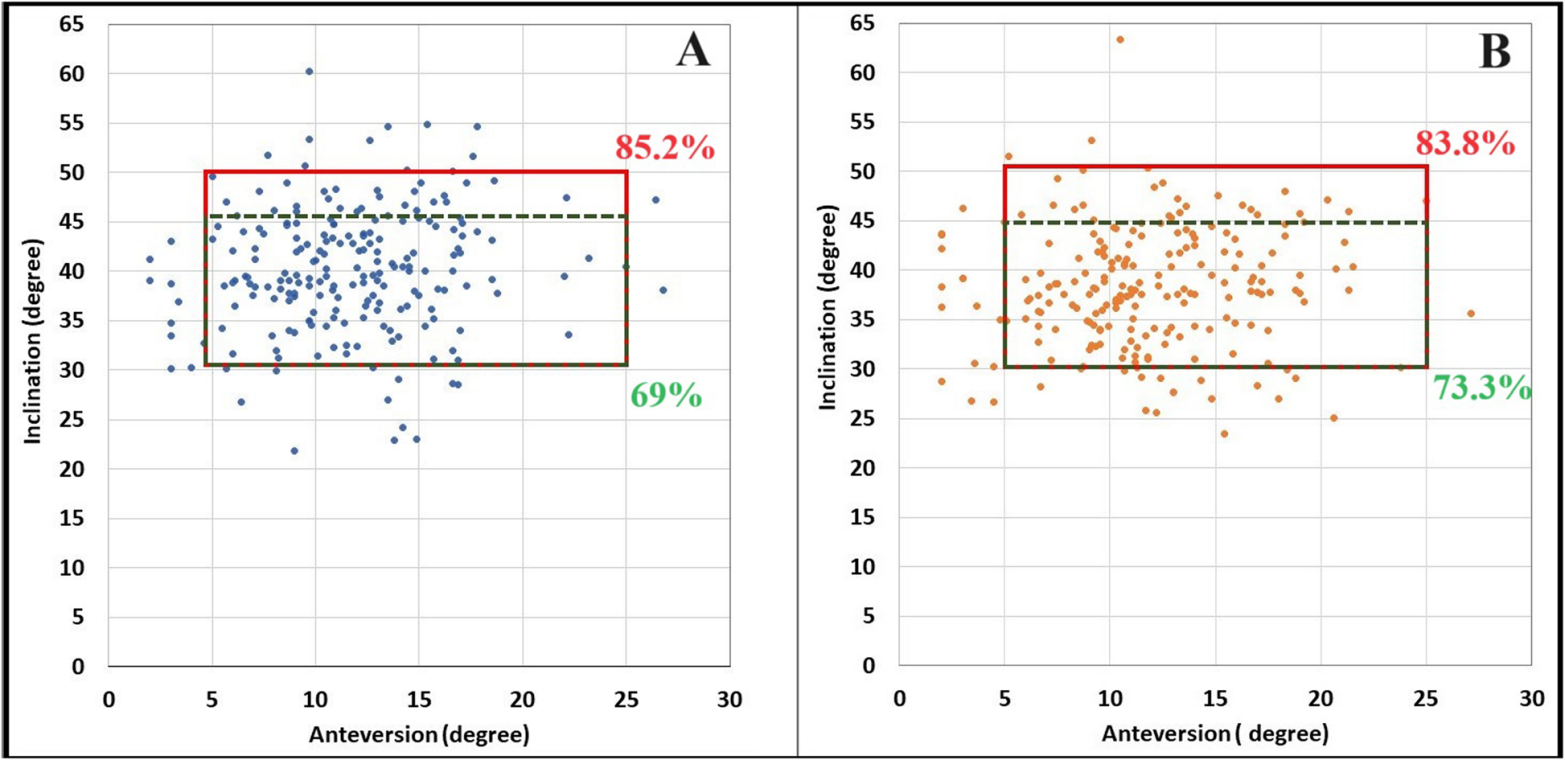
Percentage of the cups within the safe zone for both inclination and anteversion, according to Lewinnek et al. (red square), and Callahan et al. (green dotted square). **A**: right (dominant) side. **B**: left (non-dominant) side

The percentage of cups located within the safe zone for both inclination and anteversion in Group A and Group B was 85.2% vs. 83.8% and 69% vs. 73.3%, respectively according to Lewinnek and Callahan safe zone limits (Fig. 4), with no difference between the two sides in the percentage of cups placed outside the safe zone for inclination or anteversion (Table 2).

Cup position showed no difference between the types of cup fixation except for a significant difference found in the cemented cup inclination, which was higher in Group A than in Group B (40.8° ± 6.4° vs. 38.3° ± 6.3°) (*P* = 0.004), with an absolute difference of 2.5 degrees (Table 3).

**Table 3:** Association between cup fixation and positioning

	**Inclination**		*P*-Value*	**Anteversion**		*P*-Value*
	**Group A** **(dominant, right THA)**	**Group B** **(nondominant, left THA)**		**Group A** **(dominant, right THA)**	**Group B** **(nondominant, left THA)**	
**Cemented**	40.8 ± 6.4 (22.9–60.3)	38.3 ± 6.3 (23.4–63.4)	**0.004**	12.2 ± 5.1 (2.0–26.8)	11.9 ± 5.3 (2.0–27.1)	0.660
**Cementless**	39.4 ± 6.2 (21.8–54.9)	38.1 ± 5.8 (25.8–50.4)	0.12	11.2 ± 3.5 (3.0–18.6)	11.5 ± 3.7 (3.4–21.5)	0.505
P-Value**	0.121	0.754		0.08	0.51	

Data were expressed as mean ± SD (range); Bold *P*-value indicates statistically significant

^*^Independent Sample T-test compares the mean difference of malposition in both groups

^**^Independent Sample T-test compares the mean difference of malposition within the same group

## Discussion

Proper acetabular cup placement is undeniably a crucial factor dictating the functional outcomes, complication incidence, and survival rates [2–5, 33]. Acetabular cup malpositioning has been associated with increased dislocation risk, limited range of motion, accelerated wear, and eventually increased revision rates [3, 4, 34, 35]. One rarely investigated factor that could affect acetabular component positioning was the surgeon’s handedness and whether the surgery was carried out on the dominant or non-dominant side [20–24].

In the current study, an RHD surgeon showed a significant difference in cemented acetabular cup inclination when operating on the dominant vs. non-dominant side. However, the surgeon’s handedness exerted no effect on cup anteversion or the percentage of cups placed within the safe zones, leading to a partial acceptance of our hypothesis.

The surgeon’s handedness and its effect on the efficiency of training and practice have been subjects of interest for orthopedic surgeons and operators of other procedures [36–39]. Sabharwal et al. reported that 46% of their study respondents found it difficult to handle RHD instruments, and left-handed (LHD) trainees reported difficulties while being trained by RHD teaching surgeons, and they experienced significantly greater difficulty than their RHD fellow trainees (36% and 61%) (*P* < 0.001) [36].

The effect of surgeon handedness on implant positioning was studied among various orthopedic procedures. Moloney et al. attributed increased sliding hip screw failures on the left side to screw malpositioning by RHD surgeons while treating peri-trochanteric fractures [40]. During total knee arthroplasty (TKA), Liu et al. showed significantly higher femoral implant malpositioning in the sagittal plane when an RHD operated on the non-dominant side compared to the dominant side [17]. Furthermore, Mehta and Lotke reported that the outcomes in terms of knee function and pain were significantly better at six-month and one-year follow up on the dominant (377 TKAs) side compared to the non-dominant (351 TKAs) side when an RHD surgeon performed surgeries [18].

When it came to THAs, the reported results were inconsistent. Pennington et al., in 2014, were the first to investigate the relationship between surgeon handedness and acetabular cup positioning. They included four surgeons (2 RHD and 2 LHD), who alternately operated through the posterolateral and direct lateral approaches (20 THAs for each surgeon). They reported a significant difference in the cup inclination angle between the dominant and non-dominant sides, averaging 46.4° and 43.5°, respectively (*P* < 0.05) [20]. Later on, Song et al. evaluated 498 bilateral THAs performed by three RHD surgeons through a posterolateral approach (PLA) (all cementless cups). They reported a significant difference in cup inclination between the dominant and non-dominant sides (38.59° ± 6.84° and 37.50° ± 6.76°), (*P* = 0.011) [21].

Contrary to the previously mentioned results, Kong et al. evaluated 62 bilateral THAs performed by two RHD surgeons through PLA. The authors reported no differences in cup inclination on both sides (39.35 ± 5.26 vs. 40.35 ± 5.77), between the dominant and non-dominant sides, respectively, (*P* = 0.321) [22]. The same group conducted a study later on, including 102 bilateral THAs, operated by a single RHD surgeon through a direct anterior approach (DAA). They reported significantly lower cup inclination on the dominant than the non-dominant side (39.42° ± 7.19° vs. 42.61° ± 7.32°) (*P* = 0.000) [23].

It is worth noting that the absolute difference in inclination means in the current study was just 1.9 degrees. Likewise, Pennington et al. [20], Song et al. [21], and Kong et al. [23], reported a difference of 3, 1.08, and 3.2 degrees, respectively, raising the concern of clinical relevance of these radiological differences. However, considering the wide range of the differences (between − 21.4 to 22.8 degrees), and other possible contributing factors, such as anteversion, changing the hip center of rotation, leg length discrepancy, bearing surfaces, and patient factors (including body habitus and activity levels), differences in cup inclination between both sides could lead to short- (instability) as well as long-term (increase in polyethylene wear) clinical consequences [3, 41–44].

In the current study, we did not find a difference in the cup anteversion between the two sides, which could be partially ascribed to the reliance on anatomical landmark (TAL) for anteversion adjustments, which is less affected by other variables such as pelvic position change or spinopelvic relationship. Our results were consistent with the findings of Kong et al. [23], where the authors did not find anteversion differences between the two sides (15.79° ± 6.99° vs. 16.91° ± 7.49°) (*P* = 0.235). While Song et al. [21] and Kong et al. [22] reported significantly lower anteversion on the dominant side (22.01° ± 6.35° vs. 25.28° ± 7.16°) (*P* < 0.001) and (22.44° ± 8.67° vs. 24.77° ± 10.44°) (*P* = 0.043), respectively.

With regard to the percentages of cups placed within the Lewinnek safe zone, although we used manual instruments and freehand technique for cup placement, we found no differences in the percentage of cups located within the safe zones for inclination or anteversion for both sides. The same finding was reported by Kong et al. [23]. However, Song et al. reported a significant difference, where 62% and 46% were within the safe zone for the dominant and non-dominant sides, respectively (*P* < 0.001) [21]. Kong et al. included another group of 53 bilateral robotic-assisted THAs. The authors reported no difference in cup inclination or anteversion between the two sides. Furthermore, they reported that more cups were located outside the safe zone in the manual group compared to robotic-assisted THAs (70% vs. 48%, *P* = 0.001) [22].

Unlike the current study, most of the previous studies used cementless cups, in contrast to 54.3% of the cemented cups used in our study. Furthermore, we found a significant difference in cemented cup inclination between the dominant and the non-dominant sides, with an absolute difference of 2.5 degrees. This could be explained by the fact that the cemented cup position could be changed while the surgeon holds the cup until the cement settles (owing to possible hand fatigue while holding the cup inserter with steady pressure for a few minutes), unlike cementless cups, which will be seated by hammering it in the prepared acetabulum. Additionally, literature proposed several reasons trying to explain the differences between operations on the dominant and non-dominant sides, not only on joint replacement surgeries, but in different surgical specialties as well, suggesting that more powerful dominant limb and longer time to fatigue lead to more precise motor control with a subsequent quicker manipulation, and an improved capability of spatial orientation, leading to better execution of the surgical steps and handling of the instrument [45–50].

It is worth noting that surgeries in studies (including the current one) examining the effect of surgeon handedness on acetabular cup placement were performed by high-volume and experienced surgeons (per definition by Siddiqi et al. [51]) [20–24]. Accordingly, we expect this effect to amplify for low-volume and less experienced surgeons, as alluded to in the literature [2, 11, 52].

In order to avoid the added effect of surgeon handedness to cup misplacement, some strategies could be adopted by young, less experienced surgeons [5, 41]: (1) Proper preoperative planning is paramount to anticipating any other cup position-affecting factors, such as pelvic tilt and spinopelvic relations [14]; (2) Familiarity with operative theater, tables, and supporters used to secure the patient position [7]; (3) Intraoperative utilization of all possible anatomical landmarks to guide proper cup placement [5, 25]; (4) No shame in using intraoperative fluoroscopy to ensure proper acetabulum reaming and final cup position [53]; (5) Difference in handedness between trainer and trainee should be considered [36, 38, 54]; (6) Simple and cheap alternatives to expensive technologies (robotics and computer navigation), such as smartphones and related applications, helped young surgeons improve cup placement during THA [55]. Last, some surgeons suggested that DAA and supine positions are more surgeon-friendly [7, 24].

## Limitations

We admit some critical limitations of the current study. First, the study was a single surgeon, retrospective, and non-randomized one. Second, we did not report on the clinical or complication-related outcomes. Previously, Kong et al. [22, 23] reported no differences in the functional outcomes between both sides in terms of the Harris Hip Score. Furthermore, the incidence of dislocation was reported in three studies [21–23], which was stated “numerically” to be more on the non-dominant sides. Only in Song et al. study, did the authors report a statistically significant difference (7% vs. 3.2% [non-dominant vs. dominant sides]) (*P* = 0.006) [21]. Third, we did not investigate the factors behind the handedness effect when operating on both sides. However, previous studies attributed this to variations in spatial cognition and visuo-spatial ability [21, 50, 56]. Fourth, the surgeon who operated on the cases is considered experienced, with a doubtful application of the current results to limited-experience or low-volume surgeons, which affected cup positioning. Last, anteversion was evaluated radiologically, which could be criticized for less accuracy. However, the method we used proved to be equivalent to CT scans [57].

## Conclusion

Surgeon handedness could affect cup positioning, especially the inclination when surgeons use manual instruments or freehand techniques for cup placement. Furthermore, cemented cups showed variability in the inclination between the two sides. Therefore, cementless acetabular cups can offset the impact of laterality differences on cup positioning. Anteversion was less affected, possibly because it was adjusted according to fixed anatomical landmarks. Studies including left-handed surgeons and surgeons of different experience levels are paramount to the further evaluation of the effect of surgeon handedness on cup placement during THA. Future studies assessing the impact of surgeon handedness on THA functional outcomes, short-term instability, and the effect on long-term polyethylene wear rates are paramount.

## Data Availability

Data associated with this study has been deposited at https://www.kaggle.com/datasets/ahmedakhalifa/surgeon-handedness-and-acetabular-cup-placement

## Abbreviations

THA: Total hip arthroplasty
RHD: Right-handed
OA: Osteoarthritis
AVN: Avascular necrosis
RA: Rheumatoid arthritis
FNF: Fracture neck of the femur
TAL: Transverse acetabular ligament
SPSS: Statistical Package for Social Science
LHD: Left-handed
TKA: Total knee arthroplasty
PLA: Posterolateral approach
DAA: Direct anterior approach
